# Detection of Rare Germline Variants in the Genomes of Patients with B-Cell Neoplasms

**DOI:** 10.3390/cancers13061340

**Published:** 2021-03-16

**Authors:** Adrián Mosquera Orgueira, Miguel Cid López, Andrés Peleteiro Raíndo, José Ángel Díaz Arias, Beatriz Antelo Rodríguez, Laura Bao Pérez, Natalia Alonso Vence, Ángeles Bendaña López, Aitor Abuin Blanco, Paula Melero Valentín, Roi Ferreiro Ferro, Carlos Aliste Santos, Máximo Francisco Fraga Rodríguez, Marta Sonia González Pérez, Manuel Mateo Pérez Encinas, José Luis Bello López

**Affiliations:** 1 Health Research Institute of Santiago de Compostela (IDIS), 15706 Santiago de Compostela, Spain; miguel.cid.lopez@sergas.es (M.C.L.); andres.peleteiro.raindo@sergas.es (A.P.R.); jose.angel.diaz.arias@sergas.es (J.Á.D.A.); beatriz.antelo.rodriguez@sergas.es (B.A.R.); natalia.alonso.vence@sergas.es (N.A.V.); angeles.bendana.lopez@sergas.es (Á.B.L.); maximo.francisco.fraga.martinez@sergas.es (M.F.F.R.); marta.sonia.gonzalez.perez@sergas.es (M.S.G.P.); manuel.mateo.perez.encinas@sergas.es (M.M.P.E.); jose.luis.bello.lopez@sergas.es (J.L.B.L.); 2 Complexo Hospitalario Universitario de Santiago de Compostela (CHUS), Department of Hematology, SERGAS, 15706 Santiago de Compostela, Spain; laura.bao.perez@sergas.es (L.B.P.); aitor.abuin.blanco@sergas.es (A.A.B.); paula.melero.valentin@sergas.es (P.M.V.); roi.ferreiro.ferro@sergas.es (R.F.F.); 3 Complexo Hospitalario Universitario de Santiago de Compostela (CHUS), Department of Pathology, SERGAS, 15706 Santiago de Compostela, Spain; carlos.aliste.santos@sergas.es; 4 Department of Medicine, University of Santiago de Compostela, 15706 Santiago de Compostela, Spain

**Keywords:** germline, rare variant, cancer, lymphoid, B-cell, lymphoma, CLL, driver, prognosis

## Abstract

**Simple Summary:**

The global importance of rare variants in tumorigenesis has been addressed by some pan-cancer analysis, revealing significant enrichments in protein-truncating variants affecting genes such as *ATM*, *BRCA1*/*2*, *BRIP1*, and *MSH6*. Germline variants can influence treatment response and contribute to the development of treatment-related second neoplasms, especially in childhood leukemia. We aimed to analyze the genomes of patients with B-cell lymphoproliferative disorders for the discovery of genes enriched in rare pathogenic variants. We discovered a significant enrichment for two genes in germline rare and dysfunctional variants. Additionally, we detected rare and likely pathogenic variants associated with disease prognosis and potential druggability, indicating a relevant role of these events in the variability of cancer phenotypes.

**Abstract:**

There is growing evidence indicating the implication of germline variation in cancer predisposition and prognostication. Here, we describe an analysis of likely disruptive rare variants across the genomes of 726 patients with B-cell lymphoid neoplasms. We discovered a significant enrichment for two genes in rare dysfunctional variants, both of which participate in the regulation of oxidative stress pathways (*CHMP6* and *GSTA4*). Additionally, we detected 1675 likely disrupting variants in genes associated with cancer, of which 44.75% were novel events and 7.88% were protein-truncating variants. Among these, the most frequently affected genes were *ATM*, *BIRC6*, *CLTCL1A*, and *TSC2*. Homozygous or germline double-hit variants were detected in 28 cases, and coexisting somatic events were observed in 17 patients, some of which affected key lymphoma drivers such as *ATM*, *KMT2D*, and *MYC*. Finally, we observed that variants in six different genes were independently associated with shorter survival in CLL. Our study results support an important role for rare germline variation in the pathogenesis and prognosis of B-cell lymphoid neoplasms.

## 1. Introduction

B-cell lymphoid neoplasms are the most frequent hematological tumors, and they exhibit a diverse spectrum of entities with heterogeneous clinical behavior. B-cell lymphoid neoplasms are classically classified in either aggressive lymphomas (diffuse large B-cell lymphoma (DLBCL), Burkitt lymphoma, grade III follicular lymphoma, and mantle cell lymphomas) or indolent lymphomas (e.g., chronic lymphocytic leukemia (CLL), grade I/II follicular lymphoma, marginal zone lymphoma, lymphoplasmacytic lymphoma). By frequency, diffuse large B-cell lymphoma (DLBCL) is the most frequent lymphoid neoplasm, accounting for 25% of all cases of non-Hodgkin lymphoma (NHL), closely followed by CLL (19% of NHLs) and follicular lymphoma (12% of NHLs) [1].

Next-generation sequencing (NGS) technologies have deconvoluted the genomic complexity of B-cell lymphoid tumors to a great extent, revealing the most frequent molecular drivers of disease and the interplay among them. NHL cases show familial predisposition, and much of the heritability of these diseases is still unexplained [2]. Genome-wide association analysis (GWAS) have identified the existence of polymorphisms significantly associated with risk of CLL [3], DLBCL [4], and follicular lymphoma [5]. Similarly, some polymorphisms are also related with the outcome of B-cell lymphomas [6,7,8] and CLL [9], and it has also been proved that some variants cooperate with somatic events in shaping clinical outcomes of cancer patients [10]. Another source of germline variation consists of rare variants (allele frequency <0.1–1%). The global importance of such rare variants in tumorigenesis has been addressed by pan-cancer analysis, revealing significant enrichments for protein truncating variants in genes such as *ATM*, *BRCA1/2*, *BRIP1*, and *MSH6* [11]. Indeed, some of these variants predispose to cancer development through the acquisition of second somatic hits [12], such as point mutations or loss-of-heterozygosity (LOH) [13]. Additionally, germline variation can influence treatment response and contribute to the development of treatment-related second neoplasms, especially in childhood leukemia [14]. Many such rare variants in cancer-related genes have been associated with particular cancer subtypes [15,16,17], but until now little attention has been focused on the genome-wide frequency, pathogenicity, and clinical implications of rare variants in lymphoid malignancies. Rare variants in *ATM* and *CDK1* variants have been associated with CLL risk in genome-wide analysis [18], whereas evidence for the implication of infrequent events in other genes come from familial studies or single-gene analysis [19,20,21].

In this report, we performed an exploratory analysis about the frequency and distribution of rare and putatively pathogenic germline variants in the genome of several mature B-cell lymphoid neoplasms using high-throughput sequencing data produced by the International Cancer Genome Consortium (ICGC) [22]. Our results indicate the existence of multiple genes affected by highly pathogenic germline variants in the genome of these patients, some of which seem to condition patient survival.

## 2. Materials and Methods

### 2.1. Data Source

We processed germline next-generation sequencing data from 726 patients with B-cell lymphoid malignancies produced by the International Cancer Genome Consortium. Briefly, 504 cases pertained to the Spanish Chronic Lymphocytic Leukemia project, and 222 were retrieved from the German Malignant Lymphoma project. Overall, there were 504 chronic lymphocytic leukemia (CLL) or small lymphocytic lymphoma (SLL) cases (including 54 monoclonal B-cell lymphocytosis cases), 97 follicular lymphoma cases, 85 diffuse large B-cell lymphoma (DLBCL) cases, 36 Burkitt lymphoma cases, and 4 unclassified B-cell lymphoma cases. CLL control samples were derived from non-tumoral leukocytes (<2% tumor contamination), whereas lymphoma controls originated from whole blood or buffy coats checked for negative clonality analysis. Sample collection and sequencing was originally performed by the ICGC consortium.

### 2.2. Germline Variant Identification and Annotation

Most CLL germline samples (440 out of 502) were processed using exome-sequencing kits (*Agilent SureSelect Human All Exon V4 and V4+UTRs*), whereas whole-genome sequencing was performed on 262 cases, which included 62 CLL cases and the entire cohort of B-cell lymphomas included in the Malignant Lymphoma-Deutcheland (MALY-DE) project. We restricted our analysis to protein coding regions covered by the exome-sequencing kits. Variants were detected using the optimized bcbio-nextgen (version 1.1.5) pipeline [23], and the GRCh37.75 assembly was used as reference. Four different variant callers were used: freebayes (version 1.1.0.46) [24], GATK-Haplotype (GATK version 2.8) [25], Platypus (version 0.8.1.2) [26], and Samtools (version 1.9) [27], with default parameters. Homopolymers and regions with low complexity, alternative contigs, or abnormally high coverage were discarded. Similarly, we used 100bp mappability tracks in the University of Southern California (UCSC) database to filter out variants in low mappability regions. Finally, a variant was called if detected by a minimum of 2 callers and if it had a minimum genotype quality of 30 Phred and a minimum coverage depth of 10. Finally, we filtered events with variant allele fraction (VAF) <30% in order to limit possible contamination of the controls with tumor cells. Variants were annotated using dbSNP [28], 1000 Genomes [29], ExAc [30], and gnomAD [31]. Only variants with a major allele frequency (MAF) below 0.5% in any ethnic population were retained. Thereafter, we selected (1) all protein-truncating variants (PTVs): start lost, stop lost, nonsense, frameshift, splice acceptor, and splice donor variants, and (2) missense variants with pathogenicity Combined Annotation Dependent Depletion (CADD) v.14 [32] scores > 20 Phred (i.e., variants in the top 1% of predicted pathogenicity). Finally, we restricted our analysis to those genes involved in carcinogenesis, particularly in lymphomagenesis. We collected the following types of genes: (1) 162 genes involved in mendelian inherited cancer syndromes [33], (2) 723 genes included in the *Cancer Gene Census* [34], (3) 135 genes included in the TARGET database (“a database of genes that, when somatically altered in cancer, are directly linked to a clinical action” [35]), (4) 59 recurrently mutated genes in CLL [36,37], (5) 150 recurrently mutated genes in DLBCL [38], and (6) 72 recurrently mutated genes in Burkitt lymphoma [39]. The final list contained 899 non-redundant genes (Table S1). Visual analysis of all frameshift insertions and deletions was performed using *Integrative Genome Viewer* [40]. Ancestry analysis was performed using Peddy [41], which predicts ancestry using a machine learning model trained on individuals of diverse ancestries from the 1000 Genomes Project reference panel. Only 3 of the patients were of non-European ancestry (1 African, 1 South Asian, and 1 East Asian). Genes affected by 5 or more variants were annotated to the top 0.5% genes in the Frequently Mutated Genes in Public Exomes (FLAGS) database [42] in order to highlight potentially spurious discoveries. Additionally, predicted loss-of-function expected vs. observed ratios (pLOF o/e) derived from gnomAD were used to annotate these genes [43]. pLOF o/e ratio is a measure of a gene’s tolerance to protein loss-of-function variants. Genes with low pLOF o/e values are more intolerant to disrupting variants than those with high values. Finally, survival analysis was performed with cox regression. Multiple testing correction was performed with the false discovery rate (FDR) method.

### 2.3. Burden Test against Public Controls

We used *Testing Rare vAriants using Public Data* (TRAPD) software in order to compare the enrichment for putatively pathogenic variants of our cohort of patients with that of 15,708 public controls from the gnomAD version 2 whole-genome sequencing dataset [31]. Importantly, none of these controls originated from cancer studies. We performed 2 types of analysis. In the first one, a burden test was performed with all PTVs detected by the *Variant Effect Predictor* (VEP) tool [44], which determines the effect of variants on genes, transcripts, and protein sequence, as well as regulatory regions. The following types of variants were defined as PTV: splice acceptor, splice donor, stop gained, frameshift, stop lost, and start lost variants. In a second attempt, we added those variants with high impact according to SNPeff annotations [45], namely, protein–protein interaction locus variants, protein structural interaction variants (i.e., affecting variants that are in contact within the same protein), and rare amino acid variants. Only variants with a maximum allele frequency (popmax) < 0.5% in any population were selected, excluding Finnish and Ashkenazi Jewish populations and those catalogued as “Other” in gnomAD (default behavior of the “popmax” gnomAD filter). Multiple testing correction was performed with the FDR method.

We tested the association of all high-impact variants according to the annotations of VEP and SNPEff with CLL patient survival, as this was the only cohort of patients sufficiently powered to make a reliable survival analysis. We restricted our study to genes affected by high impact variants in >1% of CLL cases. We created Cox regression models for time to first treatment and overall survival, and adjustment for covariates associated with survival was performed (multivariate *p*-value < 0.2). In the first case, these were *IGHV* mutation status and tumor stage at diagnosis, whereas in the second case we adjusted for *IGHV* status and patient age at diagnosis.

### 2.4. Germline–Germline and Germline–Somatic Double Hit Event Detection

In order to identify germline double-hits, we selected concurrent rare heterozygous and putatively damaging variants affecting the same gene in the same individual. Second-hit somatic mutations were detected by comparing germline variants with somatic mutations for the same set of individuals present in the ICGC database.

### 2.5. Myeloid Clonal Hematopoiesis Filtering

Potentially mosaic somatic mutations in the blood controls due to myeloid clonal hematopoiesis of undetermined potential (CHIP) could exist. In order to assess this issue, we initially identified a list of 22 recurrently mutated genes in clonal hematopoiesis that had at least one putatively rare germline variant in the final dataset [46,47,48]. Among these genes, we analyzed if the variants were present in both the control and tumor (lymphoid) compartment, and those mutations that were not found (or found at very low VAF) in the tumoral department were catalogued as likely myeloid CHIP events.

## 3. Results

### 3.1. Rare Variants Overview

A total of 1665 rare germline variants with likely disruptive activity (CADD scores > 20 or protein truncating) were detected in 559 cancer-related genes across 693 (95.45%) patients (Table S2). Overall, the frequency of these rare and likely disrupting mutations in cancer-related genes was superior to those found in non-cancer-related genes (4.25 × 10^−3^ vs. 3.61 × 10^−3^ mutations per gene and patient). Most of these were missense variants (1559 events, 93.01%, Table 1). Interestingly, we only detected 10 likely somatic mosaic mutations among myeloid-CHIP related genes, which affected *TET2*, *DNMT3A*, *ASXL2, BCORL1*, and *PPM1D* (Table S3). These variants were removed from downstream analysis.

Overall, 113 patients (15.56%) harbored 126 PTVs in 103 different loci, which included frameshift, splice donor, splice acceptor, nonsense, stop loss, and start loss variants (Table S4). The frequency of PTVs in this gene list was notoriously superior to that observed in the remaining genes (2.11 × 10^−3^ vs. 7.33 × 10^−4^ mutations per gene and patient), suggesting an enrichment for loss of function mutations among cancer-related genes. The most frequently affected genes were *ATM* (5 cases), *SETDB1* (5 cases in a single locus), *ISX* (4 cases), and *POLQ* (4 cases).

Some of the missense variants showed a remarkable increased frequency in patients with lymphoid neoplasia compared with the non-Finnish European (NFE) gnomAD database. This was the case of the variants rs199502695 in *PRPF40B* (4 cases, 71.17 times more frequent), rs191413750 in *DOCK8* (5 cases, 55.55 times more frequent), rs377188372 in *N4BP2* (4 cases, 34.66 times more frequent), and rs146946726 in *MLLT10* (6 cases, 8.10 times more frequent).

A total of 227 different variants have also been described as pathogenic or likely pathogenic somatic mutations in cancer (Table S5). Remarkably, two out of three variants in *NOTCH1* are flagged as pathogenic somatic mutations in COSMIC. This overlap also occurred in *BCL6* (2 out of 5 variants), *PTCH1* (2 out of 4 variants), *ATM* (3 out of 14 variants), *CNOT3* (1 out of 2 variants), *DNMT1* (1 out of 2 variants), *FGFR2* (1 out of 2 variants), *JAK3* (1 out of 2 variants), *MTOR* (1 out of 2 variants), and *MDM4* (1 out of 2 variants). Furthermore, single rare variant flagged as pathogenic were also observed in *CCND2*, *CHIC2*, *CDKN1B*, *CREBBP*, *EZH2*, *FGFR3*, *JAK2*, *PRF1*, *RUNX1*, *SIRPA*, *SUFU*, *TRIP11*, and *YWHAE*.

Finally, 11 variants in homozygosity were observed, one of which (c.1642C>T in *ZCCHC8*) was present in two different patients (Table 2). Similarly, 15 patients harbored two likely functional variants in the same gene, many of which might be compound heterozygotes. Interestingly, these events were observed twice in *FAT1* and *ZFHX3*. Moreover, one homozygous nonsense variant and a germline double-hit variant case were detected in the gene *GLI1*, and one homozygous missense variant plus a germline double-hit was detected in *MYH9*. In the cases of *ARID1B* and *CBFA2T3*, the close proximity of the variants allowed us to determine that they were inherited from the same parent (Figure S1). In the remaining cases, phase data were not available.

### 3.2. Rare Variants Affecting Lymphoma Driver Genes

**Figure 1:**
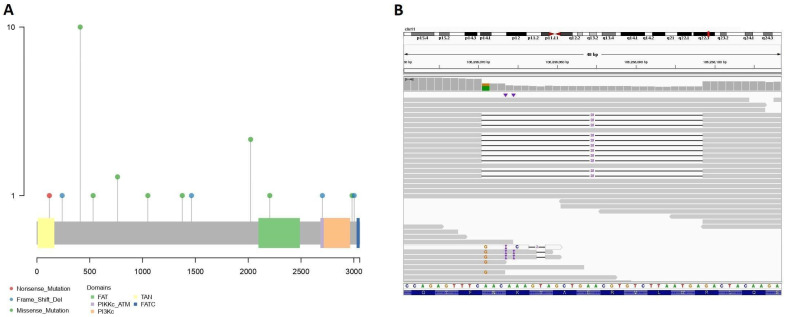
(**A**) Lollipop plot of the rare and predictively disruptive germline variants detected in the *ATM* gene. (**B**) Representation of a 28 bp frameshift deletion present in one patient.

A total of 459 different rare variants occurring 636 times in the cohort were detected across 143 driver genes of lymphomagenesis extracted from the literature. These events affected 415 patients (57.16%) (Table S6). The most commonly mutated genes were *ATM* (25 cases, Figure 1A,B), *BIRC6* (24 cases), *SPEN* (15 cases), *ZNF292* (13 cases), *MGA* (12 cases), *BAZ2A* (12 cases), *NCOR1* (11 cases), *GNA13* (10 cases), and *WDR7* (10 cases) (Table 3).

Various variants also affected other drivers of lymphomagenesis, such as *ARID1A* (9 cases), *CHD1* (9 cases), *MECOM* (9 cases), *NOTCH1* (7 cases), *SETD2* (6 cases), *ARID1B* (5 cases), *BLC6* (5 cases), *CTCF* (5 cases), *EP300* (5 cases), *JAK3* (5 cases), *NOTCH2* (5 cases), *MET* (5 cases), *MYC* (5 cases), *TCF3* (5 cases), and *CHD2* (4 cases). Finally, infrequent variants in *TRAF2* were detected in three patients, whereas those of *CNOT3*, *ID3*, *IKZF3*, *MTOR*, *POT1*, and *STAT5B* occurred in two patients each, and those of *ASXL1*, *BRAF*, *CARD11*, *CCND2*, *CCND3*, *CREBBP*, *DTX1*, *ETV6*, *EZH2*, *KRAS*, *MCL1*, and *TCL1A* were detected in just one case each. Notably, both variants in *SAMDH1* were PTVs (a frameshift and a nonsense event) (Table S7).

### 3.3. Rare Variants Affecting Genes Involved in Cancer Syndromes with Germline Inheritance

A total of 84 genes associated with inherited cancer syndromes were affected by a total of 372 occurrences of 225 different rare variants (Table S8), of which 19 were PTVs and affected 22 patients (3%). In total, 131 variants were observed in genes linked with autosomal dominant syndromic cancer, affecting 168 patients. Among these, the most frequently mutated genes were *TSC2* (22 cases), linked to tuberous sclerosis; *APC* (16 cases), linked to hereditary colon cancer; and the DNA polymerase *POLE* (16 cases), involved in predisposition to multiple cancers (Table 3). Similarly, 94 variants in 32 genes linked to autosomal recessive cancer were observed, which affected 149 patients. The most commonly affected among these were *ATM* (25 cases), *NBN* (12 cases), *BLM* (12 cases), *DOCK8* (12 cases), and *WRN* (12 cases) (Table 3).

Some of these variants were labelled as pathogenic in *ClinVar* (Table S6). This included a missense variant in *MITF* (rs149617956, 7 cases), a missense variant in *GBA* (rs76763715, 6 cases), a missense variant in *MUTYH* (rs34612342, 2 cases), two missense variants in *SERPINA1* (rs61761869 and rs28931570, 3 cases), a missense variant in *NBN* (rs61754966, 1 case), and a frameshift deletion in *BRCA2* (rs397507591, 1 case). Likely pathogenic variants were detected in *APC* (missense variant, rs139196838, 1 case), *BRIP1* (frameshift insertion, rs878855150, 1 case), and *MET* (missense variant, rs34589476, 1 case).

Interestingly, 95 previously undescribed variants were detected, and these were particularly frequent in *ATM* (4 missense variants, 1 nonsense variants, and 2 frameshift deletions, including a 28 base pair deletion), *EXT1* (2 missense variants and 1 splice donor variants), *MET* (2 missense variants and 1 frameshift deletion), *MITF* (1 missense variants in 2 patients and 1 missense variants in 1 patient), *DOCK8* (3 missense variants), *MSH6* (2 missense variants and 1 nonsense variant), *SMARCA2* (3 missense variants), *SOS1* (3 missense variants), *TRIM37* (3 missense variants), and *WRN* (1 missense, 1 nonsense, and 1 splice gain variant).

### 3.4. Rare Variants in Genes of the Cancer Gene Census and TARGET Databases

A total of 327 occurrences of 208 rare variants in 95 different genes linked to therapy were identified. These affected 247 patients (34.02%) (Table S9). The most recurrently affected genes were *ATM* (25 cases), *TSC2* (22 cases), *APC* (16 cases), *ROS1* (16 cases), and *JAK2* (11 cases) (Table 3). Among *Cancer Gene Census* genes, 1346 events were detected in 947 different loci (Table S10), with the most recurrent ones being those in *CLTCL1* (24 cases), *CSMD3* (21 cases), *MYH9* (20 cases), *ANK1* (19 cases), *TPR* (19 cases), *MYH11* (18 cases), *PTPN13* (18 cases), and *POLQ* (17 cases) (Table 3). Some genes were enriched in PTV variants, particularly *POLQ* (4 out of 10 different variants), *AKAP9* (3 out of 10), *TSHR* (2 out of 3), and *ISX* (2 out of 2). Furthermore, one pathogenic (rs113994096, 5 patients) and one likely pathogenic (rs138929605, 1 patient) missense variants in the DNA polymerase *POLG* were also discovered.

### 3.5. Differential Distribution of Rare Variants and Association with Patient Survival

We did not identify any gene significantly enriched in rare variants in CLL vs. B-cell lymphoma cases (Fisher’s test, FDR < 5%). Nevertheless, we discovered that some variants were only detected in one subgroup. For example, the missense variant rs1800729 in *TSC2* was exclusively present in CLL (eight cases), and the missense variant rs139075637 in *POLE* was exclusively present in non-CLL B lymphoid tumors (seven cases). Notably, both variants were found to have higher frequency in non-Finish Europeans than in other populations according to gnomAD data (allele frequencies of 0.40% and 0.17%, respectively). Therefore, further analysis needs to be performed in order to confirm these findings and rule-out population substructure biases.

**Figure 2:**
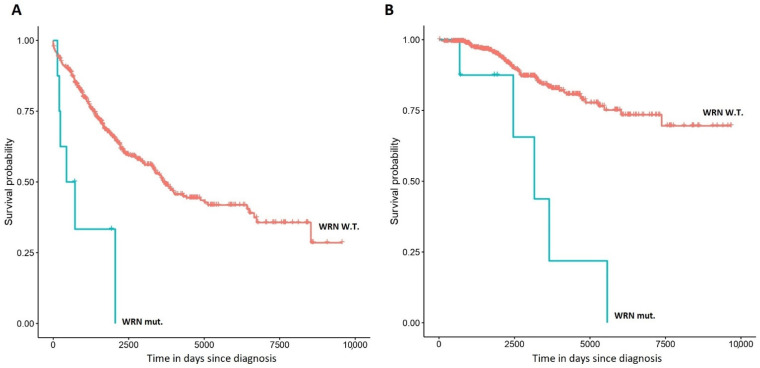
Kaplan–Meier plots representing the association of rare variants in *WRN* with time to first treatment (**A**) and overall survival (**B**) in chronic lymphocytic leukemia (CLL). Variants considered in this plot are represented in Table 4.

Thereafter, we tested if rare variants could be associated with adverse patient outcomes. Due to the heterogeneity of the dataset and sample size limitations, we restricted our analysis to CLL cases, and considered variants present in at least 1% of cases. Interestingly, rare variants in the DNA helicase *WRN* (8 cases, Table 4) were significantly associated with shorter overall survival (Cox *p*-value 1.16 × 10^−4^, *q*-value 0.01, Hazard Ratio (HR) (2.35, 14.59); Figure 2B). Indeed, such association was independent of age at diagnosis and CLL/MBL status (*p*-value 1.97 × 10^−7^, HR (5.03, 35.48)). Moreover, these variants were also linked to shorter time to first treatment (Cox *p*-value 6.15 × 10^−4^, HR (1.85, 9.48); Figure 2A), which remained significant after adjusting for age at diagnosis and CLL/MBL status (*p*-value 1.69 × 10^−3^, HR (1.64, 8.48)). These patients tended to harbor high-risk karyotype anomalies in the tumor cells: 11q deletion (three cases, one as an isolated anomaly, one co-occurring with 13q deletion, and one co-occurring with three other karyotype anomalies), 17p deletion (one case, co-occurring with a 18p deletion), 8q deletion (one case, co-occurring with 21q gain), and 6q deletion (one case, co-occurring with 13q deletion). The most frequent variant was rs78488552 (six out of eight cases), which has its highest frequency in non-Finish Europeans (0.49%).

Rare variants in *ATM* have been previously associated with CLL risk [18]. Curiously, no association with survival could be observed in this analysis. As *ATM* is enriched in missense variants [18], we restricted the analysis only to patients with truncating events (four cases), and discovered that these few cases had a significantly shorter overall survival (*p*-value 0.02, HR (1.28, 21.53)).

### 3.6. Association of Rare Germline Variants with Somatic Mutations

Concurrent rare and likely disruptive germline variants and somatic mutations were detected in 17 cases (Table 5). Co-occurring mutations in CLL affected *GNA13*, *KMT2D*, *LRP1B*, *MUC16*, and *SPEN*. Additionally, co-occurring mutations in B-cell lymphomas were found in *CSMD3* (grade I follicular lymphoma), *EP300* (DLBCL), *FAT1* (DLBCL), *HIST1H1E* (grade I follicular lymphoma), *KMT2D* (DLBCL), *MCL1* (grade IIIa follicular lymphoma), *MSH6* (grade IIIa follicular lymphoma), *MYC* (DLBCL), *PIM1* (grade I follicular lymphoma), *RNF213* (DLBCL), and *SIN3A* (grade IIIb follicular lymphoma). Additionally, we observed a germline mutation in *ATM* co-occurring with a 11q copy neutral loss of heterozygosity that induced loss of the reference allele in a CLL patient.

### 3.7. Burden Test of High Impact Variants Using Public Controls

**Figure 3:**
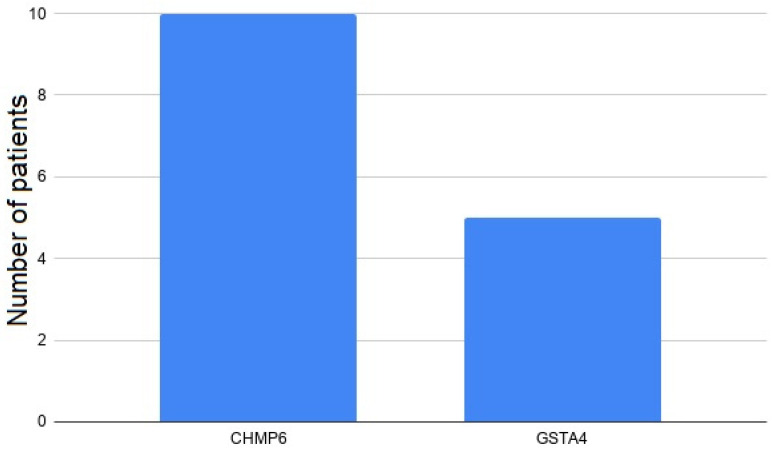
Frequency of highly dysfunctional variants within genes identified in the burden test.

Germline variants with high functional impact were selected for association with risk of B-cell neoplasms using the burden test against public whole-genome sequencing controls. Briefly, this analysis tests if the cumulative frequency of variants affecting each gene in a cohort is significantly different from that of a control cohort. We first analyzed all PTVs detected by VEP, and afterwards we added all variants with high impact consequences according to SNPeff. We selected these variants because they are the most potentially pathogenic. As a result, two genes were significantly enriched in high-impact variants among patients affected by B-cell lymphoid neoplasms (*q*-value < 0.1) (Figure 3, Table S11). Overall, we identified 2 different variants affecting 15 different cases (Table S12, Figure 3): rs746495175 in *CHMP6* (a splice acceptor variant) and rs557844606 in *GSTA4* (an inframe deletion within a structural interaction domain). Importantly, inflation statistics were low (ƛ = 0.93 and 0.83 for the VEP-only and VEP + SNPeff models). Additionally, there was an enrichment of *CHMP6* variants in lymphoma vs. CLL patients (Fisher’s *p*-value 0.02, *q*-value 0.04).

### 3.8. Association of High Impact Variants with Patient Survival

High impact variants in four genes were independently associated with shorter CLL patient survival (*q*-value < 0.1; Table 6). These genes were *M1AP* (Figure S2), *GNLY*, *FLYWCH1*, and *PIK3C2G*. Variants in another gene (*PLA2G7*) were also suggestively associated with short survival (*q*-value 0.11). Conversely, we did not detect variants in any gene associated with either time to first treatment or earlier age at diagnosis.

## 4. Discussion

Approximately 8% of cancer patients are affected by pathogenic germline variants, which confer a strong hereditary component [49]. Interestingly, growing evidence indicates that such variants can modulate cancer evolution and prognosis. For example, truncating variants in genes of the angiogenesis and DNA repair pathways predispose to the development of metastatic disease in prostate cancer [15]. Therefore, we reasoned that the analysis of such variants in patients affected by B-cell lymphoid neoplasms could shed new clues about their pathogenesis and prognostication. Indeed, our results indicate an increased frequency of highly disruptive rare variants in two genes of B-cell lymphoid tumor patients: *CHMP6* and *GSTA4*. Notably, both genes are involved in cell survival regulation under oxidative stress. *GSTA4* mediates glutathione-dependent elimination of 4-hydroxynonenal, which is an important product of peroxidative degradation of arachidonic acid [50]. At the same time, *CHMP6* encodes a member of membrane repair dependent on endosomal sorting complexes required for transport (ESCRT)-III, which inhibit ferroptosis (a form of cell death triggered by iron accumulation and lipid peroxidation) [51].

Additionally, our data indicate a significant contribution of these high-impact rare variants to CLL survival, as we found significant or suggestive associations of five genes with overall survival. Notably, all the affected genes play a role in oncogenic pathways. For example, *M1AP* is involved in meiosis progression, and recent evidence supports a role as a positive regulator of the oncogene *MYC* [52]. *GNLY* encodes granulysin, a protein located in cytotoxic granules of Natural Killer and T-cells. Interestingly, it has been observed that granulysin triggers cancer cell apoptosis through caspase-dependent and independent mechanisms in hematological B-cell neoplasms, and therefore it plays a central role in immune-related mechanisms of tumor development and progression [53]. Similarly, *FLYWCH1* and *PIK2C2G* regulate oncogenic signaling through WNT/β-catenin and phosphoinositide-3-kinase pathways, respectively [54,55]. Finally, *PLA2G7* encodes a lipoprotein-associated phospholipase that regulates epithelial-mesenchymal transition, and it is associated with the development of metastatic disease in solid organ cancer [56]. Overall, our pioneer results indicate that these mutations can act as true drivers of disease progression and treatment failure, even though they are not recurrently mutated in the somatic line.

In a different approach, we focused our research on the detection of rare and likely disruptive mutations (both PTVs and non-PTVs) in a set of genes involved in cancer pathways, and particularly in lymphoid neoplasms. The collective high frequency of these rare germline variants in cancer genes poses a challenge for personalized genomics, as many of these are probably non-functional, whereas others play a pathogenic or prognostic role. We identified recurrent highly pathogenic variants affecting important drivers of hematological cancer (*ATM* [57]), epigenetic regulators (*ISX* [58] and *SETDB1* [59]), and mediators of DNA replication (*POLQ* [60]). Recurrent variants were also observed in drug targets, particularly in the crizotinib targets *ALK*, *MET*, and *ROS1*, as well as the everolimus target *TSC2*, which suggests new therapeutic strategies for these patients [61,62,63]. Additionally, several variants were previously catalogued as pathogenic (such as the E318K variant in the transcription factor *MITF* [64]); others affected strong mediators of inherited predisposition to lymphomas (i.e., *DOCK8*, *EXT1*, *MSH6*, and *SOS1* [65,66,67,68]), while others have been flagged as pathogenic somatic mutations in cancer, such as *NOTCH1* R912W [69,70] and *CNOT3* E20K [71,72]. Importantly, we observed that variants in the DNA helicase *WRN* were significantly associated with shorter overall survival and time to first treatment in CLL. *WRN*-mutated CLL cases tended to harbor high-risk karyotypic anomalies, suggesting an increased genomic instability [73] mediated by altered DNA repair mechanisms [74].

Germline–germline or germline–somatic “*double-hit*” events were identified in cancer driver genes. Germline–germline double-hit events were detected in 28 cases (3.85% of cases), and curiously five genes affected more than one patient, including the Hedgehog signaling gene *GLI1* [75] and the homeobox tumor suppressor *ZFHX3* [76]. Additionally, germline-somatic “*double hit*” events occurred in 17 cases (2.34%). Notably, this phenomenon affected common drivers of lymphomagenesis, such as the oncogenes *MYC* and *PIM1* [77,78]; the tumor suppressors *ATM*, *FAT1*, *KMT2D*, and *MSH6* [79,80,81]; the histone acetyltransferase *EP300* [82]; the histone gene *HIST1H1E* [83]; the transcriptional regulator *SIN3A* [84]; the NOTCH pathway member *SPEN* [85]; and the apoptotic proteins *GNA13* and *MCL1* [86,87].

This study has several limitations. First, some background heterogeneity could exist between Spanish CLL and German lymphoma populations. Although the current knowledge does not support heterogeneity between Spanish and German lymphoma populations, it must be considered that fine-scale population structure at extremely fine scales has been documented, even within neighboring Iberian populations [88]. Secondly, many relevant oncogenes and tumor suppressors were very rarely mutated, and the interpretation of these variants in terms of survival will need the sequencing of thousands of cases. Additionally, the presence of mosaic somatic mutations in the controls due to clonal hematopoiesis could have led to some false positives. In this line, we observed that only a minority of variants in genes associated with CHIP were likely somatic events, but nevertheless our results should be taken with caution among this group of genes. Finally, another limitation arises from the heterogeneity and limited sample size of the B-cell lymphoma dataset, which dissuaded us from making a survival analysis in such cases.

## 5. Conclusions

Our results indicate the existence of multiple genes affected by highly pathogenic germline variants in the genomes of patients with B-cell neoplasms, including a significant enrichment for high impact rare variants in two genes related to oxidative stress regulation. Additionally, the association of some variants with shorter survival, along with the disruptive nature of some others, points towards new functional, prognostic, and therapeutic implications. Finally, the elevated number of rare and likely pathogenic variants in cancer genes supposes a challenge for personalized genomics, and future analysis integrating more layers of biological information and other types of cancers are envisaged in order to clarify their benign or pathogenic role.

## Figures and Tables

**Table 1:** Distribution of the selected variant types present in the cohort.

Variant Type	Frequency
Missense	93.50%
Stopgain	2.40%
Frameshift deletion	1.60%
Frameshift insertion	0.80%
Splice donor	0.80%
Splice acceptor	0.40%
Nonframeshift deletion	0.20%
Stoploss	0.10%
Nonframeshift insertion	0.10%
Startloss	0.10%

**Table 2:** List of all homozygous and germline double-hit rare and putatively dysfunctional variants detected across 726 patients with B-cell lymphoid neoplasms.

Case ID	Zygosity	Gene	Variant Type	rs ID
147	Germline double-hit	*ARID1B*	missense	rs1378351788, rs200808642
1416	Germline double-hit	*ATM*	missense and frameshift deletion	No rs IDs
1196	Germline double-hit	*CBFA2T3*	missense	rs143704547, rs561624190
4167381	Germline double-hit	*EPPK1*	missense	rs144123426, no rsID
544	Germline double-hit	*FAT1*	missense	rs377498159, no rsID
1078	Germline double-hit	*FAT1*	missense	rs201279606, rs201751862
4190784	Germline double-hit	*GLI1*	missense	rs200306754, no rsID
1260	Germline double-hit	*IL6ST*	missense	rs191125510, rs199939306
1565	Germline double-hit	*MYH9*	missense	rs56200894, no rsID
772	Germline double-hit	*NCOR1*	missense	rs118021690, no rsID
4159421	Germline double-hit	*PIM1*	missense	No rsIDs
284	Germline double-hit	*RNF213*	missense	rs202143169, rs141301945
63	Germline double-hit	*WRN*	missense	rs78488552 and no rsID
757	Germline double-hit	*ZFHX3*	missense	rs148334947, rs147016640
1191	Germline double-hit	*ZFHX3*	missense	rs148334947 and no rsID
4126692	Homozygote	*AKAP9*	missense	rs61757664
1298	Homozygote	*ERBB3*	missense	rs55699040
1309	Homozygote	*GLI1*	nonsense	No rsIDs
308	Homozygote	*MYH9*	missense	rs139134727
325	Homozygote	*MYO5A*	missense	rs147898420
4115001	Homozygote	*NTRK3*	missense	No rsIDs
1568	Homozygote	*PDZRN3*	missense	rs141385664
7	Homozygote	*SFRP4*	missense	rs147145122
1437	Homozygote	*SYNE1*	missense	No rsIDs
396	Homozygote	*TSC1*	missense	rs118203504
1052;1295	Homozygote	*ZCCHC8*	missense	rs150057798

**Table 3:** List of the most recurrently affected genes by rare and predictively disruptive germline variants. Genes in the top 0.5% of the FLAGS list are indicated, as well as predicted loss-of-function (pLOF) observed vs. expected ratio (o/e ratio) along with its 90% confidence interval.

Gene	No. Cases	FLAGS Top 0.5%	pLOF o/e Ratio
*FAT3*	32	Yes	0.18 [0.13–0.25]
*SYNE1*	31	Yes	0.37 [0.33–0.42]
*FAT1*	29	Yes	0.34 [0.27–0.43]
*ATM*	25	No	0.6 [0.51–0.71]
*BIRC6*	24	No	0.07 [0.04–0.1]
*CLTCL1*	24	No	0.8 [0.66–0.98]
*ZFHX3*	23	Yes	0.08 [0.05–0.14]
*TSC2*	22	No	0.02 [0.01–0.07]
*CSMD3*	21	No	0.23 [0.18–0.3]
*MYH9*	20	No	0.04 [0.02–0.09]
*TPR*	19	No	0.06 [0.04–0.11]
*LRP1B*	19	Yes	0.26 [0.21–0.32]
*ANK1*	19	No	0.1 [0.06–0.17]
*EPPK1*	19	Yes	0.96 [0.78–1.2]
*KMT2D*	19	Yes	0.07 [0.04–0.1]
*PTPN13*	18	No	0.52 [0.42–0.64]
*MYH11*	18	No	0.22 [0.16–0.3]
*POLQ*	17	No	1.05 [0.9–1.22]
*KMT2C*	17	Yes	0.08 [0.06–0.12]
*MUC4*	16	No	0.84 [0.7–0.99]
*APC*	16	No	0.1 [0.06–0.16]
*ROS1*	16	No	0.95 [0.82–1.12]
*POLE*	16	No	0.52 [0.42–0.64]
*NBEA*	16	No	0.04 [0.02–0.07]
*SPEN*	15	No	0.03 [0.01–0.07]
*SETDB1*	15	No	0.11 [0.06–0.2]
*LPP*	15	No	0.32 [0.19–0.56]
*FAT4*	15	Yes	0.12 [0.08–0.18]

**Table 4:** Rare and likely functional mutations in *WRN* among CLL patients. One patient had two concurrent variants (indicated with an asterisk *). Nucleotide and amino acid changes induced by each variant are provided.

Chromosome	Position	rsID	REF	ALT	CLL Cases	Mutation Type
8	30922465	.	T	G	1 *	c.390T>G; p.Asn130Lys; missense variant ENST00000298139 transcript, exon 5/35 SIFT score: 0.01 (deleterious) Polyphen score: 0.913 (probably damaging)
8	30922580	.	G	A	1	c.504+1G>A; splice donor variant ENST00000298139 transcript, exon 5/34
8	30954292	rs569266355	A	G	1	c.1907A>G; p.Tyr636C; missense variant ENST00000298139 transcript; exon 17/35 SIFT score: 0 (deleterious) Polyphen score: 0.99 (probably damaging)
8	31012237	rs78488552	C	G	6 *	c.3785C>G; p.Thr1262Arg; missense variant ENST00000298139 transcript, exon 32/35 SIFT score: 0 (deleterious) Polyphen score: 0.959 (probably damaging)

**Table 5:** Cases of co-occurring somatic mutations and rare germline variants in the same gene. Marked with an asterisk is an event where a rare and likely disruptive germline variant in *ATM* coexisted with a loss-of-heterozygosity (LOH) at 11q that deleted the wild-type allele.

Gene	Case ID	Diagnosis
ATM *	155	CLL
*GNA13*	381	CLL
*KMT2D*	372	CLL
*LRP1B*	122	CLL
*MUC16*	1267	CLL
*SPEN*	830	CLL
*EP300*	4122063	DLBCL
*KMT2D*	4175941	DLBCL
*MSH6*	4109808	DLBCL
*MYC*	4107559	DLBCL
*PIM1*	4102009	DLBCL
*RNF213*	4109808	DLBCL
*CSMD3*	4111337	Follicular lymphoma
*FAT1*	4136095	Follicular lymphoma
*HIST1H1E*	4144951	Follicular lymphoma
*MCL1*	4159421	Follicular lymphoma
*SIN3A*	4139696	Follicular lymphoma

**Table 6:** Association results of high-impact variants with overall survival in CLL.

GENE	FDR	Lower 95% CI HR	Upper 95% CI HR	CLL Cases
*M1AP*	1.61 × 10^−2^	2.88	32.52	7
*GNLY*	4.04 × 10^−2^	2.63	50.45	6
*FLYWCH1*	7.54 × 10^−2^	1.8	19.32	10
*PIK3C2G*	7.74 × 10^−2^	1.94	37.94	6
*PLA2G7*	0.11	1.64	29.43	6

## Data Availability

Data for this study were downloaded from the International Cancer Genome Consortium repository.

## Supplementary Materials

The following are available online at https://www.mdpi.com/2072-6694/13/6/1340/s1, Figure S1. IGV plots for rs1378351188 (G > A) and rs200808642 (G > T) in *ARID1B* and rs143704547 (G > T) and rs561624190 (A > C) in *CBFA2T3*. Figure S2. Kaplan–Meier plot representing the association of high impact rare variants in *M1AP* with overall survival in CLL. Table S1. Candidate gene list. The table represents the relationship between each candidate gene and each gene list included in the analysis. Table S2. Annotated list of all rare and likely disruptive variants detected across 726 patients with B-cell lymphoid neoplasms. Table S3. Myeloid CHIP-related genes affected by rare variants in this study. The number of known variants and the number of likely somatic mosaic events detected is indicated in the corresponding columns. Table S4. Annotated list of all rare and likely disruptive protein-truncating variants across 726 patients with B-cell lymphoid neoplasms. Table S5. List of all filtered rare germline events overlapping known somatic mutations in the COSMIC database. Table S6. List of all rare and likely disruptive variants in known drivers of B-cell lymphoid tumors detected across 726 patients with B-cell lymphoid neoplasms. Table S7. List of all filtered rare germline events included in the ClinVar database. Table S8. List of all rare and likely disruptive variants in genes linked to syndromic cancer detected across 726 patients with B-cell lymphoid neoplasms. Table S9. List of all rare and likely disruptive variants in genes of the TARGET database across 726 patients with B-cell lymphoid neoplasms. Table S10. List of all rare and likely disruptive variants in genes of the Cancer Gene Census database across 726 patients with B-cell lymphoid neoplasms. Table S11. Burden test results for the VEP-only and VEP + SNPEff models. Results with *q*-value < 0.1 are shown. Table S12. High-impact variants affecting genes significantly enriched in patients with B-cell lymphoid neoplasms.
