# Supplementary material for: Detection of Rare Germline Variants in the Genomes of Patients with B-Cell Neoplasms
Source: Cancers (Basel). 2021 Mar 16;13(6):1340. doi: 10.3390/cancers13061340 (PMC8001490; doi:10.3390/cancers13061340)
Supplement: Supplementary file 1 [file cancers-13-01340-s001.zip › cancers-1094718-supplementary-final/Supplementary Figure S1-S2.docx]

Detection of Rare Germline Variants in the Genomes of Patients with B-Cell Neoplasms

Adrián Mosquera Orgueira, Miguel Cid López, Andrés Peleteiro Raíndo, José Ángel Díaz Arias, Beatriz Antelo Rodríguez, Laura Bao Pérez, Natalia Alonso Vence, Ángeles Bendaña López, Aitor Abuin Blanco, Paula Melero Valentín, Roi Ferreiro Ferro, Carlos Aliste Santos, Máximo Francisco Fraga Rodríguez, Marta Sonia González Pérez, Manuel Mateo Pérez Encinas and José Luis Bello López


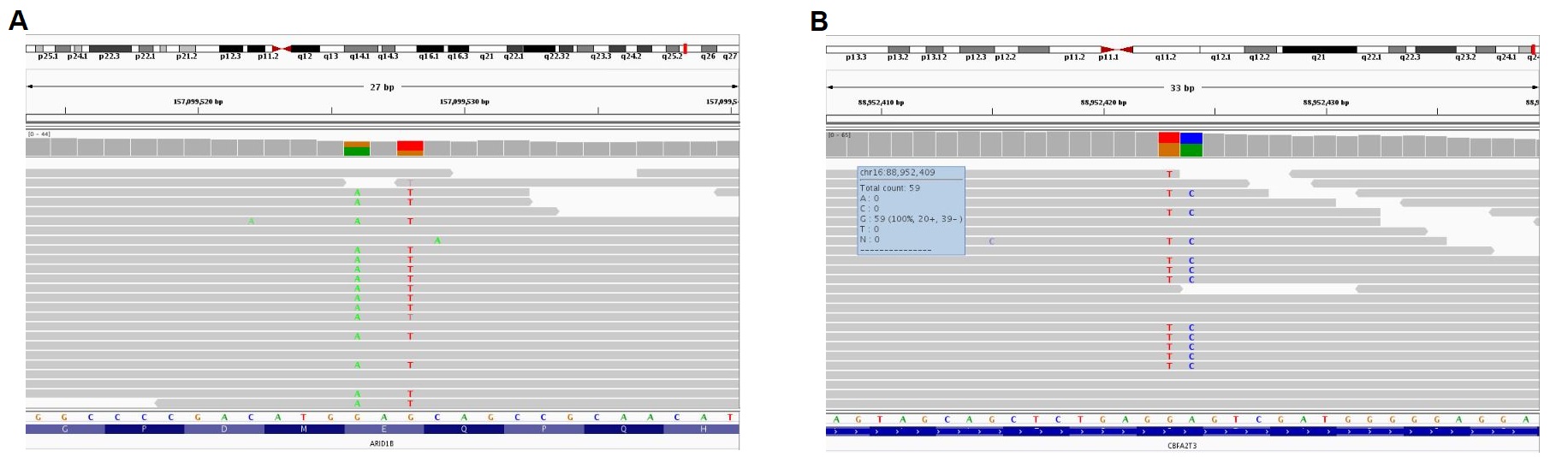


**Figure S1.** IGV plots for rs1378351188 (G>A) and rs200808642 (G>T) in ARID1B and rs143704547 (G>T) and rs561624190 (A>C) in CBFA2T3.


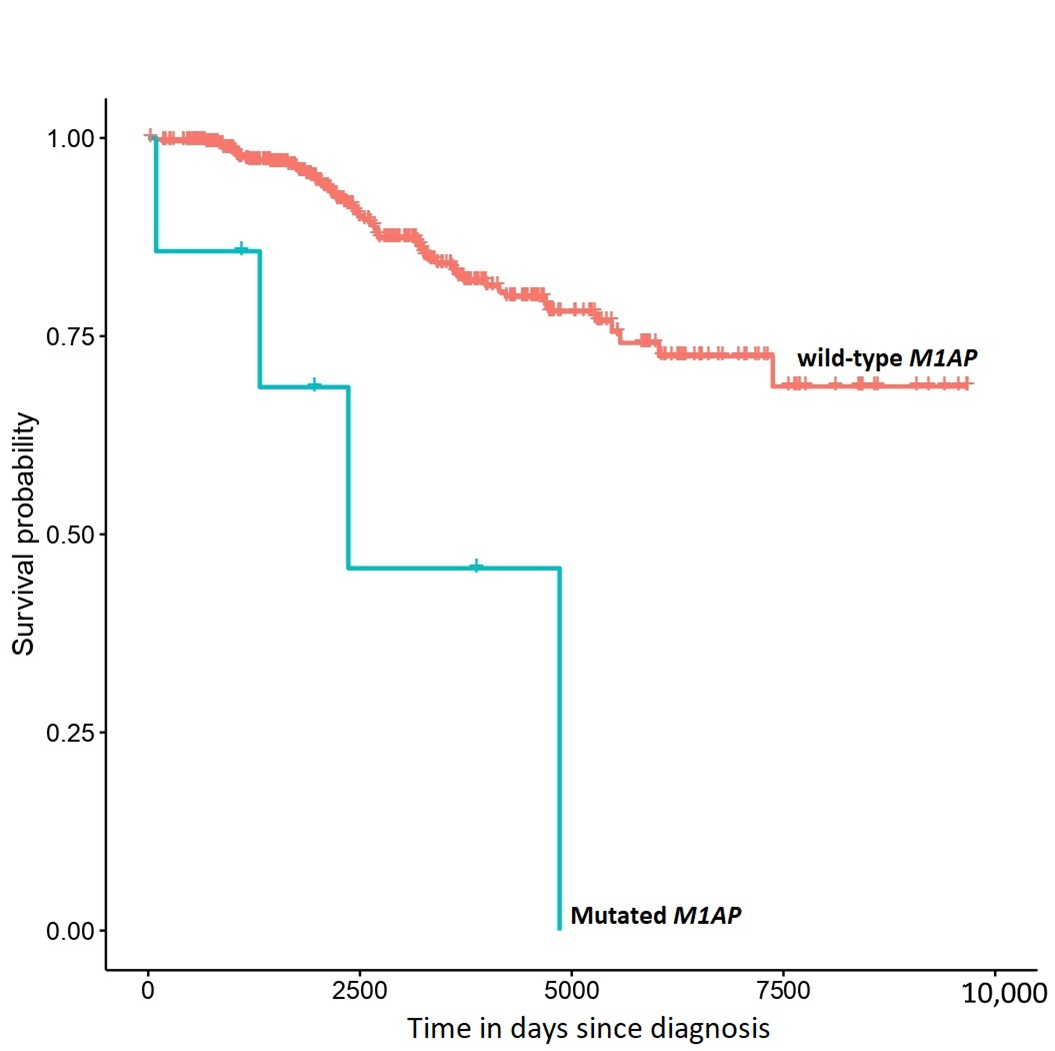


**Figure S2.** Kaplan–Meier plot representing the association of high impact rare variants in M1AP with overall survival in CLL.
